# Stress Intensity Factor Assessment for the Reinforcement of Cracked Steel Plates Using Prestressed or Non-Prestressed Adhesively Bonded CFRP

**DOI:** 10.3390/ma14071625

**Published:** 2021-03-26

**Authors:** Emilie Lepretre, Sylvain Chataigner, Lamine Dieng, Laurent Gaillet

**Affiliations:** SMC Laboratory, MAST Department, Gustave Eiffel University, Allée des Ponts et Chaussées, 44344 Bouguenais, France; sylvain.chataigner@univ-eiffel.fr (S.C.); lamine.dieng@univ-eiffel.fr (L.D.); laurent.gaillet@univ-eiffel.fr (L.G.)

**Keywords:** CFRP bonded reinforcement, mild steel, stress intensity factor, finite element analysis, pre-stressed CFRP

## Abstract

The use of adhesively bonded carbon fiber reinforced polymer (CFRP) materials to reinforce cracked steel elements has gained widespread acceptance in order to extend the lifespan of metallic structures. This allows an important reduction of the stress intensity factor (SIF) at the crack tip and thus a significant increase of the fatigue life. This paper deals with the assessment of the SIF for repaired cracked steel plates, using semi-empirical analysis and finite element analysis. Metallic plates with only one crack originating from a center hole were investigated. Virtual crack closure technique (VCCT) was used to define and evaluate the stress intensity factor at crack tip. The obtained modeling results are compared with experimental investigations led by the authors for different reinforcement configurations including symmetrical and non-symmetrical reinforcement, normal modulus and ultra-high-modulus CFRP plates, and pre-stressed CFRP plates. Results show that finite element model (FEM) analysis can obviously simulate the fatigue performance of the CFRP bonded steel plates with different reinforcement configurations. Moreover, a parametric analysis of the influence of the pre-stressing level was also conducted. The results show that an increase of the pre-stressing level results in an increase of the fatigue life of the element.

## 1. Introduction

Next to corrosion, fatigue is the most common problem influencing the durability and thus the safety of steel structures. Fatigue cracks mainly occur in stress concentration areas and quickly propagate, leading to catastrophic structural failure. The use of adhesively bonded CFRP (carbon fiber reinforced polymer) materials is now a widespread repairing method for structural reinforcement and reparation of cracked steel structures [1]. This method provides significant benefits (CFRP materials are lightweight materials with good fatigue and corrosion resistance) and avoids some drawbacks compared to conventional methods (e.g., additional welded or bolted plates).

Bonding CFRP material improves the performance of the repaired structures by sharing the load and by amplifying the crack closure phenomenon. The final effect is a decrease of the effective stress intensity factor (SIF) at the crack tip and consequently a decrease of the rate of fatigue crack growth.

Previous studies on the reinforcement of steel elements by bonded CFRP have shown an increase of the fatigue lifetime compared to un-repaired steel members [2,3,4,5,6,7]. It was shown that an increase of the tensile stiffness of the CFRP reinforcement, by using a double-sided bonding configuration and/or by using CFRP material with a higher Young modulus leads to a greater reduction of the SIF at the crack tip and thereby increases the fatigue lifetime. It was also demonstrated that, for better results, the bonding area of CFRP should cover the overall location of the crack [4,8].

In addition to the above researches, some studies have investigated the effectiveness of pre-stressed CFRP plates in increasing the fatigue lifetime [9,10,11,12,13,14,15,16]. Adding pre-stressing force in the CFRP material allows using a more significant portion of the strength of the material. Moreover, by applying an adequate level of pre-stress to the CFRP laminate, the effective SIF at crack tip can be reduced below the SIF threshold and therefore lead to a full stop of the crack propagation [11,14,15,16,17,18,19,20].

In this paper, reinforcement of part of riveted metallic bridges by CFRP material was investigated. Fatigue cracks in riveted structures mainly initiate from rivet holes in elements in tension (mode-I fatigue crack). For a high number of rivets (more than four rivets in a line), Yin et al. [21] showed that the fatigue resistance of a riveted joint (with and without pre-tension of the rivets) is similar to that of a plate with a center hole. To date, researchers have conducted various investigations (both experimental and numerical) on the use of FRP to repair symmetrical through-thickness cracks [2,3,4,6,8,9,12,13,15,22,23]. However, in riveted assembly, only one fatigue crack typically originates from the center hole (un-symmetrical through-thickness crack). For that reason, a distinguishing feature of our study is to consider small-scale metallic specimens with only one fatigue crack emanating from a center hole.

In a preceding study led by the authors [24,25,26], the efficiency of different CFRP repair configurations, in order to prolong the fatigue lifetime of both mild steel and wrought iron un-symmetrical cracked steel plates, was proven. Non-pre-stressed and pre-stressed normal modulus (NM) CFRP laminates as well as ultra-high modulus (UHM) CFRP laminates were studied, with both single and double-sided repairing configurations. In riveted structures, due to the presence of multi-plate components, the accessibility to both sides of the cracked element is often impossible. Therefore, the single-sided reinforcement was more particularly studied. As the efficiency of such reinforcement was experimentally proven, the next step was to develop reliable analysis tools to predict the behavior of the repaired elements.

In this paper, a semi-empirical analysis and a finite element analysis were proposed in order to evaluate the stress intensity factor reduction ratio for the various studied reinforcement configurations. Virtual crack closure technique (VCCT) was used and several SIF values were analyzed in order to give a proper evaluation of the fatigue crack propagation for each reinforcement configuration. A good fitting was observed between experimental and numerical results emphasizing the importance of selecting the appropriate SIF value. Finally, the numerical model was used to lead a parametric analysis of the effect of the pre-stressing level in the CFRP laminate. The results showed that a higher pre-stressing level led to a more significant reduction in SIF. Nevertheless, the non-symmetric reinforcement configuration led to a secondary bending that reduced the reinforcement effect.

## 2. Summary of the Experimental Studies on CFRP-Reinforced Cracked Steel Plates

Previous experimental works on cracked metallic plates made of S235 carbon steel were performed in order to investigate the effectiveness of different CFRP reinforcement configurations (see [26]).

Specimens were 510 mm long, 90 mm wide and 10 mm thick with a 20 mm diameter hole. An initial 0.6 mm wide notch (at the edge of the hole) was made using the wire eroding technique. From this mechanical notch, the specimen was then pre-cracked using a fatigue loading procedure. The aim of fatigue pre-cracking is to provide a sharpened fatigue crack similar to a natural crack. In our case, the pre-cracking procedure was used to obtain a crack length of 7 mm from the edge of the hole, which ensured that the effect of the initial notch was removed from the specimen.

**Figure 1:**
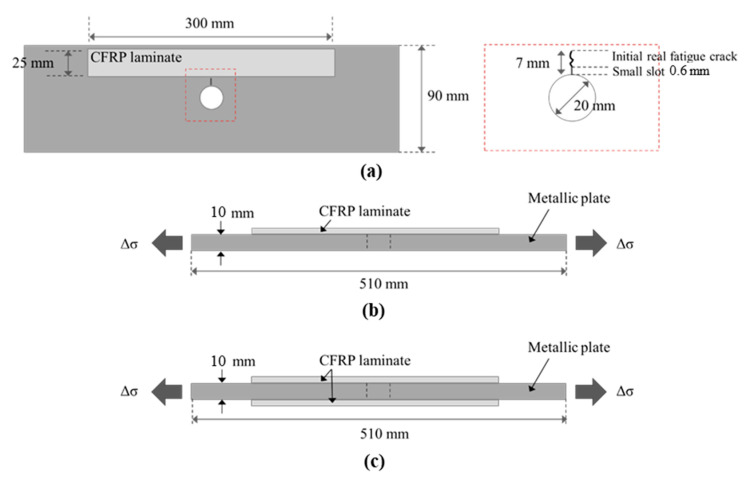
Configuration of CFRP (carbon fiber reinforced polymer) reinforcement for cracked metallic plates (not to Scheme 165. GPa (in fiber’s direction) and a thickness of 1.2 mm, and the UHM (ultra-high modulus) pultruded CFRP laminate had a Young modulus of 460 GPa (in fiber’s direction) and a thickness of 2.3 mm. The material properties of the CFRP laminates, the associated adhesives and the steel plates are listed in Table 1. (**a**) plan view, (**b**) section view of single-side repaired specimens, (**c**) section view of double-side repaired specimens.

For all repaired samples, the CFRP laminate (bonded either on one side or on both sides of the cracked plate) was bonded at a distance of 10 mm from the edge of the hole, thus taking account of the possible presence of the rivet head. The geometry and dimensions of the samples are given in Figure 1.

The specimens were tested under tensile cyclic loading until complete failure of the samples. A stress ratio of 0.1 and a frequency of 10 Hz were considered, corresponding to an applied stress range in the nominal section of the steel plates varying from 10 MPa to 100 MPa. Four reinforcement configurations were tested, including single-sided repair with non-pre-stressed NM CFRP laminates (S_NM_NP_SS), single-sided repair with pre-stressed NM CFRP laminates (S_NM_P(10kN)_SS), double-sided repair with non-pre-stressed NM CFRP laminates (S_NM_NP_DS) and single-sided repair with non-pre-stressed UHM CFRP laminates (S_UHM_NP_SS). Un-strengthened specimens (reference one), named S_R, were also tested for comparison.

The experimental results are summarized in Table 2. The fatigue life increase ratio corresponds to the ratio between the fatigue life of the strengthened specimen and the fatigue life of the un-strengthened specimens (reference specimens). The fatigue lifetime was increased by a factor ranging from 1.27 to 2.27, depending on the reinforcement configuration. For single-sided repaired specimens, the greatest increase in fatigue life was obtained for pre-stressed NM CFRP plates and UHM CFRP plates.

**Figure 2:**
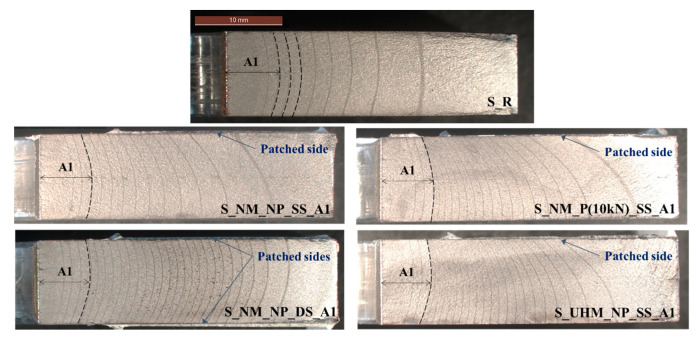
“Beach marks” obtained for both un-strengthened and strengthened S235 carbon steel specimens with different reinforcement configurations.

The “beach marking” technique was used to follow the real crack front shape and to measure its size. This technique consists of cyclically reducing the applied stress range for a small number of cycles during the propagation stage of the fatigue crack. The reduction in the applied stress range causes changing of the stress intensity factor (SIF) value at the crack tip and thus a decrease of the crack growth rate, which leaves visible marks on the crack surface of the material. Observation of real crack size and shape in the thickness of the specimen is then possible after complete failure of the steel plates. More details about the measurement of the crack propagation for all reinforced specimens can be found in [26]. Figure 2 shows the “beach marks” obtained for all samples (strengthened and un-strengthened ones).

For all specimens (un-strengthened and strengthened ones), a curved crack front was observed. This phenomenon, called tunneling effect, is due to the difference of stress states in the thickness of the plates. For the thick specimen, the stress state near the center was close to plane-strain condition, while near the side surface, the stress state was close to plane-stress condition [27]. Therefore, crack closure phenomena would be much more significant in a small area near the specimen surface. According to Fiordalisi [28] and Branco et al. [29], this small region ranges from approximately 1 to 2 mm in the thickness of the plate.

Regarding the single-sided repair specimens, another phenomenon appeared due to the non-symmetric configuration. This phenomenon was the secondary bending (shift of the neutral axis) generated when the external load was applied. The additional moment worsened the crack opening on the un-patched side of the plate and therefore caused the observed non-uniform trough-thickness crack profile.

All these observations supported the fact that the SIF at crack tip, which governs the crack propagation, experienced a large variation through the thickness of the plate, particularly in the case of single-sided repaired specimens. These observations were also reported in other studies on composite patch repair [30,31,32,33]. It is therefore understandable that the prediction of the behavior of repaired structures by CFRP bonded involved an analysis of the SIF modification, taking into account these phenomena.

## 3. Assessment of Modified Stress Intensity Factor for Repaired Steel Plate

### 3.1. Stress Intensity Factor (SIF) of Un-Repaired Specimens (Reference Ones)

For a 2D plate, the theoretical mode-I SIF at the crack tip can be expressed as Equation (1) [34]:

(1) $$\Delta K=F\cdot \Delta \sigma \cdot \sqrt{\left(\pi \cdot a\right)}$$

where Δ*σ* is the applied stress range, *a* is the edge crack length, and *F* is a geometry correction factor (depending on the part’s geometry, the type of loading and the crack length).

**Figure 3:**
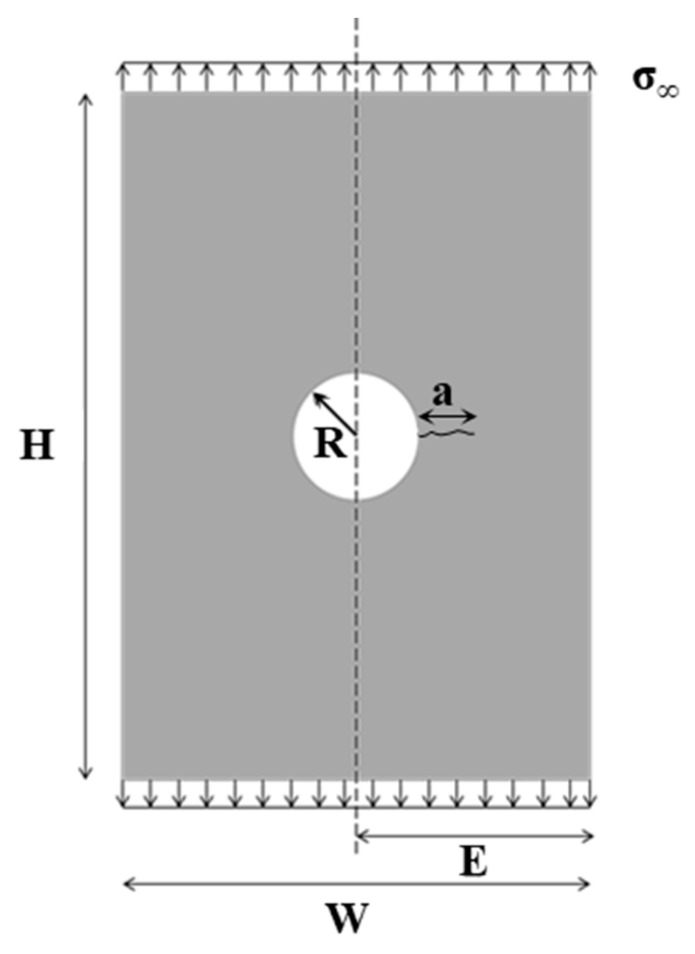
Dimensions considered for the 2D stress intensity factor (SIF) analytical solution.

For a non-repaired 2D cracked steel plate with one crack emanating from a center hole (see Figure 3), the geometry correction factor can be expressed through a classical solution available in literature (handbooks). 

In our study, the Palmberg solution [35] was adopted, Equations (2)–(5):

(2) $$F=\;F_{OH}^{1}\cdot F_{W}^{1}\cdot F_{H}^{1},$$

(3) $$F_{OH}^{1}=\;\left[\frac{1}{0.539+1.93\cdot \left(\frac{a}{R}\right)+2\cdot {\left(\frac{a}{R}\right)}^{2}}+\frac{\lambda +2}{2}\right]\cdot \sqrt{\frac{\lambda +1}{2}\cdot \left[1+\frac{a}{R}\cdot \frac{\lambda^{3}}{5}\right]}\;\lambda =\;\frac{1}{1+\left(\frac{a}{R}\right)},$$

(4) $$F_{W}^{1}=\sqrt{\sec\left(\frac{\pi \cdot R}{W}\right)\cdot \sec\left(\frac{\pi }{2}\cdot \frac{2R+a}{W-a}\right),}$$

(5) $$F_{H}^{1}=1$$

where *a* is the edge crack length, *R* is the radius of the hole and *W* the width of the plate.

The equations presented above are valid for a 2D plate (plane-stress assumption). Nevertheless, for a 3D plate, a triaxial state of stress exists. According to Bakker [36], using a 2D solution to calculate SIF results in an error by a factor of ${\left(1-\nu^{2}\right)}^{-0.5}$. Thus, for our study, this error was taken into account by multiplying by ${\left(1-\nu^{2}\right)}^{-0.5}$ the 2D SIF analytical solution (Equation (1)).

### 3.2. Determination of the Modified Stress Intensity Factor (SIF) Using Semi-Empirical Analysis

In order to determine the experimental modified mode-I SIF for S235 carbon steel reinforced by bonded CFRP a semi-empirical method based on the Paris law [37] and the James–Anderson method [38] was used:

1. First, the Paris law parameters, *C* and *m* (which only depends on the material properties) are determined from a fatigue test on the reference specimens (index R, see Equation (6)):
  (6) (dadN)R=C·ΔKm
  where a corresponds to the crack length measured at mid-thickness of the plate (maximum crack length), N corresponds to the number of cycles, and $\Delta K=K_{max}-K_{min}$ is the SIF range at crack tip obtained from the classical solution previously given.
  From the experimental results, values of 9.86 × 10^−14^ and 3.04 (*∆K* in MPa.mm^1/2^ and *da/dN* in mm/cycle) were respectively obtained for C and m.
2. Then, from the fatigue tests on the CFRP-reinforced specimens (index CFRP), the crack growth rate is determined (see Equation (7)):
  (7) (dadN)CFRP=f(a)
  For double-sided repaired specimens, the crack length corresponds to the measure at mid-thickness of the plate, while for single-sided specimens, the crack length corresponds to the measure at the un-patched surface of the plate (maximum crack length).
3. Finally, the Paris law is used for the CFRP-reinforced specimens (considering the same parameters, *C* and *m*), which allows determining the modified SIF, (ΔK)_CFRP_, for each reinforcement configuration (see Equation (8)):
  (8) (ΔK)CFRP=[1C·(dadN)CFRP]1m

The value of material constants C and m are the same for the fatigue analysis of the repaired specimens. That means that the effect of the CFRP patch only acts as a reduction of the stress intensity factor.

Thus, due to the CFRP reinforcement, it is clear that the SIF at crack tip will be modified. This modification can be expressed by means of a reduction factor, *F_CFRP_*, defined as the ratio between the modified SIF for each reinforced steel plate (obtained numerically or experimentally) and the SIF for un-reinforced specimens (reference specimens), Equation (9):

(9) $${\left(\Delta K\right)}_{CFRP}=F_{CFRP}\cdot {\left(\Delta K\right)}_{R}$$

The objective of this study is thus to propose a reliable finite element model able to determine the reduction factor *F_CFRP_* for each reinforcement configuration.

### 3.3. Determination of the Modified Stress Intensity Factor (SIF) Using Finite Element Model

The numerical simulation was done using the commercial nonlinear finite element code MSC Marc Mentat (MSC Software, version 2018.1.0) [39]. Three-dimensional element models of all specimens were built based on the configuration of the tested specimens (see Figure 1).

**Figure 4:**
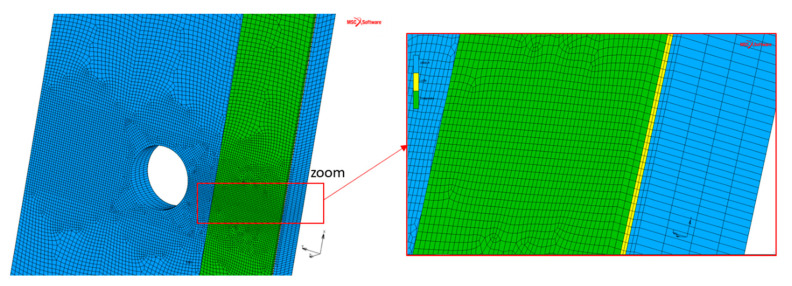
Finite element model of the reinforced plate.

The steel plate, the CFRP laminate and the adhesive layer were modeled by using hexahedral mesh elements (8-node elements). The meshing was refined in the vicinity of the crack tip due to the existence of high stress concentration, while bigger elements were used near the plate edges, as shown in Figure 4. The element type and size were chosen through a sensitivity analysis in order to ensure that the finite element model (FEM) was able to make reliable predictions of the SIF. In this way, a mesh size of 0.5 mm in the width of the steel plate in the vicinity of the crack tip was considered. A through-thickness straight crack was set at the edge of the hole corresponding to the initial crack.

Bond condition (glue contact) was set at the interface between CFRP laminate and the adhesive layer. Regarding the interface between the steel substrate and the adhesive layer, glue contact was also considered but a glue deactivation option was set at the nodes on the crack front line. With the glue deactivation option, the considering nodes were doing regular contact instead of glued contact. The debonding failure at the adhesive–plate interface was thus not considered in this study.

The steel plate and the adhesive layer were modeled as isotropic linear elastic materials, while the CFRP plate was modeled as an orthotropic linear elastic material. The input mechanical properties for steel plate, CFRP laminates (NM and UHM) and their associated adhesives (named A and B, respectively) are given in Table 3.

Cycling tensile loading was applied to one end of the steel plate (cyclic face load) while the other end was fixed to zero degrees of freedom (no displacement or rotation allowed) in order to imitate the specimen under test conditions. The minimum and maximum applied stresses of the cycle were, respectively, 10 MPa and 100 MPa.

Linear elastic fracture mechanics (LEFM), by means of the virtual crack closure technique (VCCT), was used to evaluate the strain energy release rate, called *G* [40].

**Figure 5:**
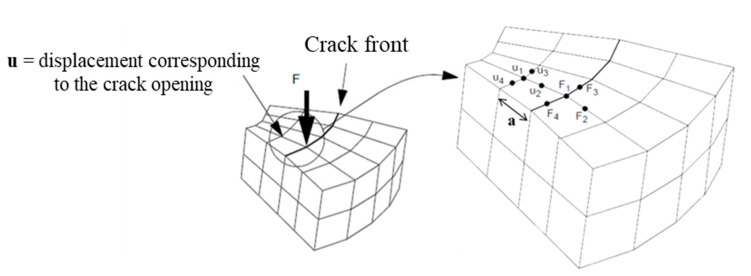
Determination of the energy release rate in 3D modeling [39].

For a 3D modeling with MSC Marc Mentat software the strain energy release rate is determined using the Equation (10):

(10) $$G=\frac{F_{1}\cdot u_{1}+\;F_{2}\cdot u_{2}+\;\frac{1}{2}\cdot \left(F_{3}\cdot u_{3}+F_{4}\cdot u_{4}\right)}{2\cdot a}$$

where *F_i_* is the constraint force at crack tip node, *u_i_* is the displacement at the node immediately behind the crack tip and *a* is the crack length increment (see Figure 5).

Finally, as the S235 carbon steel is a linear elastic material, the strain energy release rate, G, can be related to the SIF, K, by the Equation (11):

(11) $$K=\;\sqrt{\left(G\cdot E^{*}\right)}\;E^{*}=E\;\;\;\;(\mathrm{for}\;\mathrm{plane}-\mathrm{stress}\;\mathrm{condition})\;E^{*}=\frac{E}{\left(1-\nu^{2}\right)}\;\;(\mathrm{for}\;\mathrm{plane}-\mathrm{strain}\;\mathrm{condition})$$

where E and ν are, respectively, the elastic modulus and the Poisson’s ratio of the steel plate.

## 4. Results

### 4.1. Validation of the Model

**Figure 6:**
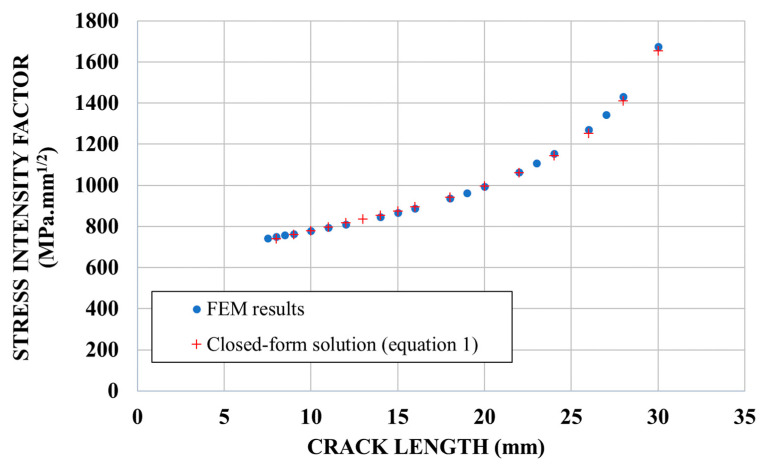
SIF (Stress Intensity Factor) versus crack length for un-strengthened specimens.

Figure 6 shows the SIF versus the crack length for un-strengthened specimens, obtained from FEM results and from the classical solution used in Equation (1). The crack length and SIF are the values at mid-thickness of the plate. We can observe a good correlation between the closed-form solution of SIF and the FEM results (error less than 2%), which validates the model.

**Figure 7:**
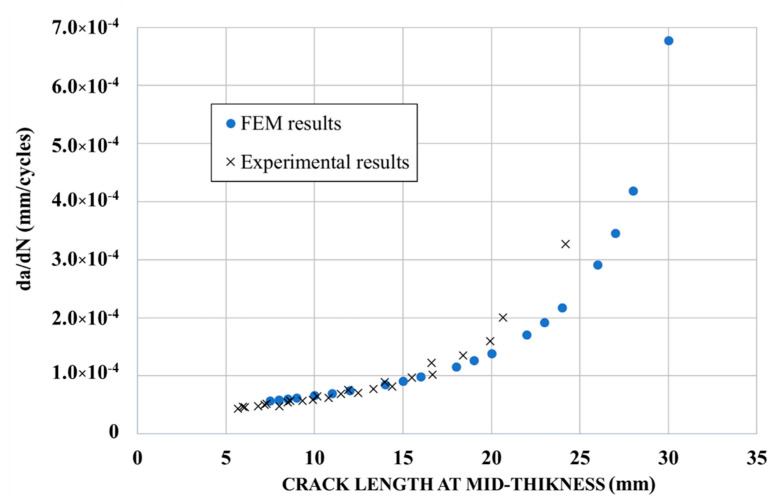
Crack growth curve for un-strengthened specimens.

The SIF values from FEM results were then used to calculate the crack propagation by using the parameters *C* and *m* (Paris law). The crack growth curves obtained from FEM results and from experiments are shown in Figure 7.

Figure 7 shows a good prediction of the crack propagation using the FEM (good correlation with the experimental results). From a crack length of 20 mm and above, an increase of the crack propagation rate was observed experimentally, corresponding to the end of the stable crack propagation field of the Paris law (as the inelastic region at the crack tip extends, the elastic stress analysis becomes inexact, and thus Equation (1) becomes no longer valid).

**Figure 8:**
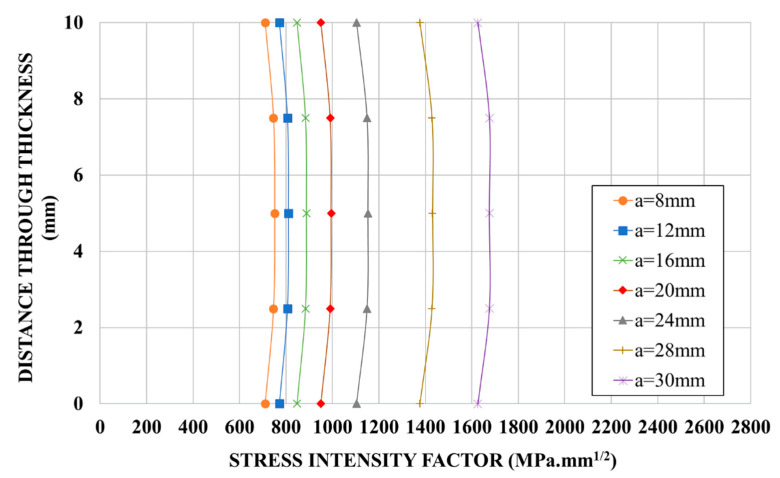
Distribution of SIF along crack front for un-strengthened specimens obtained from FEM (finite element model) results.

Figure 8 shows the distribution of SIF along the crack front obtained from the FEM results for un-strengthened specimens (the indicated crack length was measured at mid-point). It is interesting to note that the distribution shape is compatible with the crack front shape observed experimentally (curved crack front observed in Figure 2) with a maximum stress intensity factor obtained at mid-thickness of the plate.

Therefore, the non-uniform distribution observed in Figure 8 highlights the importance of choosing the correct value of SIF for the calculation of the crack propagation (for which only one value of SIF per crack length is needed). This particular point will be discussed hereinafter regarding the reinforced specimens.

### 4.2. Reinforced Specimens

#### 4.2.1. Distribution of the Stress Intensity Factor (SIF)

**Figure 9:**
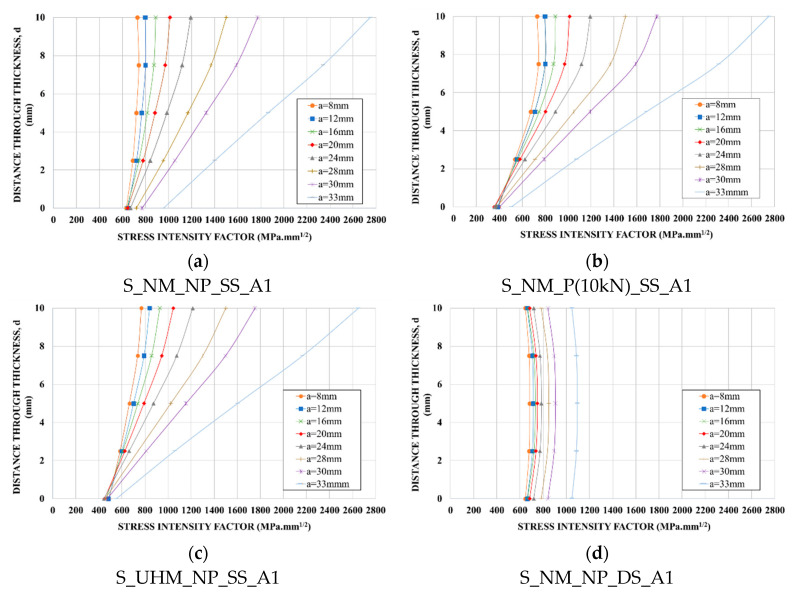
Distribution of SIF along crack front for strengthened specimens obtained from FEM results: (**a**) normal modulus (NM)-non-prestressed single-sided repair specimen; (**b**) NM-pre-stressed single-sided repair specimen; (**c**) ultra-high modulus (UHM)-non-prestressed single-sided repair specimen; (**d**) NM-non-prestressed double-sided repair specimen.

**Figure 10:**
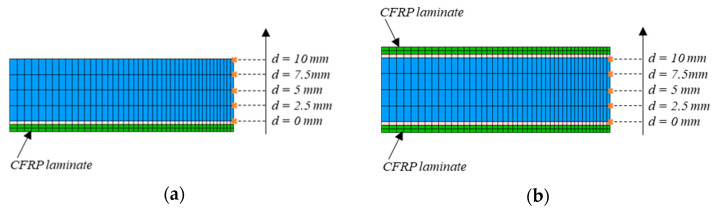
Measuring points through thickness of the steel plate used for SIF analysis with FEM: (**a**) single-sided repair specimen; (**b**) double-sided repair specimen.

Figure 9 shows the distribution of the SIF along the crack front for each repaired configuration obtained from FEM results. The measuring points through thickness of the steel plate used for SIF analysis with FEM are represented in Figure 10.

**Figure 11:**
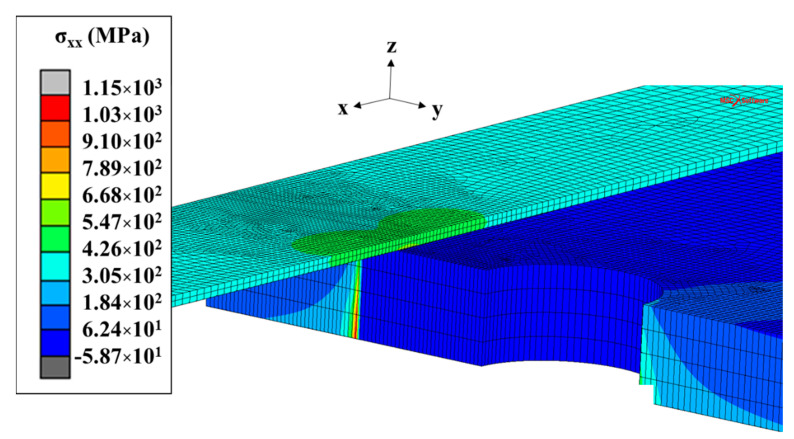
Distribution of the normal stress in a single-sided repair specimen.

For single-sided repair specimens, we can observe that the maximum SIF value shifted toward the free side of the plate. Actually, the load transfer between the steel plate and the CFRP laminate mainly occurred in the patched side, as we can see in Figure 11 (the stress at crack tip was smaller in the reinforced side). This led to a significant decrease of SIF for the crack tip immediately under the patched surface (see Figure 9a–c).

This reduction was more significant for specimens reinforced by pre-stressed NM CFRP laminate and UHM CFRP laminate, for which the non-uniform SIF distribution along the crack front was more pronounced. These observations are compatible with the experimental ones regarding the crack front shape (see Figure 2).

For double-sided repaired specimens, the distribution of SIF was symmetric about the mid-thickness of the steel plate. As for the un-strengthened specimen, the maximum SIF was obtained at mid-thickness of the plate. However, we can observe, for the same crack length, that the SIF at mid-thickness was much smaller for the double-sided reinforced specimen than for the un-strengthened specimen, indicating a significant slow-down of the crack propagation.

#### 4.2.2. Determination of the SIF Value for Each Crack Length

In order to determine the crack propagation (crack length versus number of cycles), a single value of SIF for each crack length was needed. For single-sided reinforced specimens (showing a significant non-uniform distribution of SIF along crack front), a concern existed regarding the choice of the adequate value of the SIF for each crack front.

**Figure 12:**
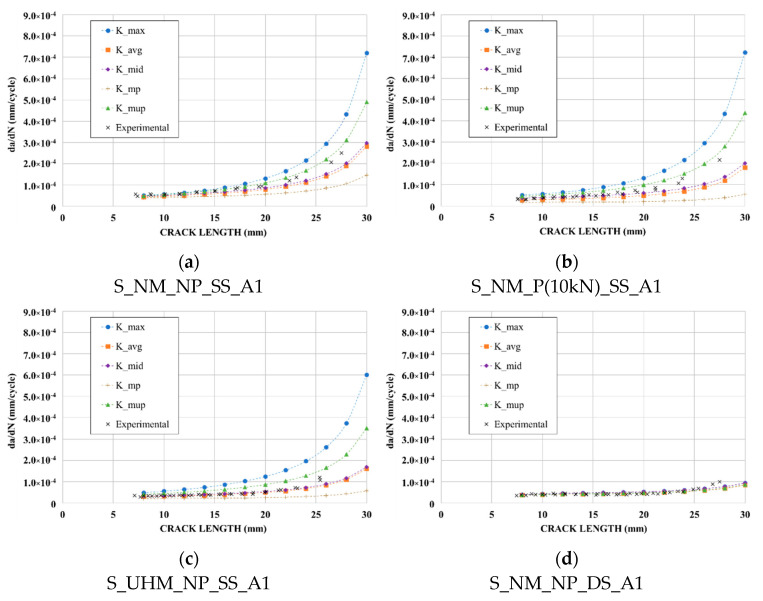
Comparison between experimental and predicted crack growth curves using averaged FEM results: (**a**) Normal Modulus-non-pre-stressed single-sided reinforced specimen; (**b**) Normal Modulus-pre-stressed single-sided reinforced specimen; (**c**) Ultra High Modulus-non-pre-stressed single-sided reinforced specimen; (**d**) Normal Modulus-non-pre-stressed double-sided reinforced specimen.

Figure 12 shows the crack growth curves obtained from both experimental and FEM results for all reinforced specimens. Regarding FEM results, different values of SIF for each crack length were considered, namely:

- the maximum value of SIF along the crack front (*K_max*);
- the average of all values along the crack front (*K_avg*);
- the mid-point value in thickness (*K_mid*);
- the average of values from mid-point to patched side (*K_mp*);
- the average of values from mid-point to un-patched side (*K_mup*).

As a reminder, for experimental results, the crack length was measured at mid-thickness of the steel plate for double-sided repaired specimens and at the un-patched side for single-sided reinforced specimens (corresponding in both cases to the maximum crack length that governed the crack propagation).

For double-sided reinforced specimens, there was no significant difference between the results obtained from all considered SIF values. For these specimens, the average of all SIF values along the crack front (*K_avg*) could be used to determine an accurate crack growth rate.

For single-sided reinforced specimens, Figure 12 highlights the importance of choosing the adequate numerical value of SIF in order to assess the fatigue lifetime. As expected, a determination of the SIF at the un-patched surface (*K_max*) led to a too conservative assessment of the fatigue life, while a determination of the SIF at the patched surface (*K_mp*) of the plate led to a non-conservative assessment of the fatigue life. These observations have already been made in others studies [31,41].

It appears that, until a crack length of 20 mm, the use of the value (*K_mid*), and to a lesser extent the use of (*K_avg*), allowed a good prediction of the crack propagation for all single-sided reinforced specimens. Above this length, the end of the validity of the Paris law field was reached and the crack growth prediction using LEFM became inaccurate.

**Figure 13:**
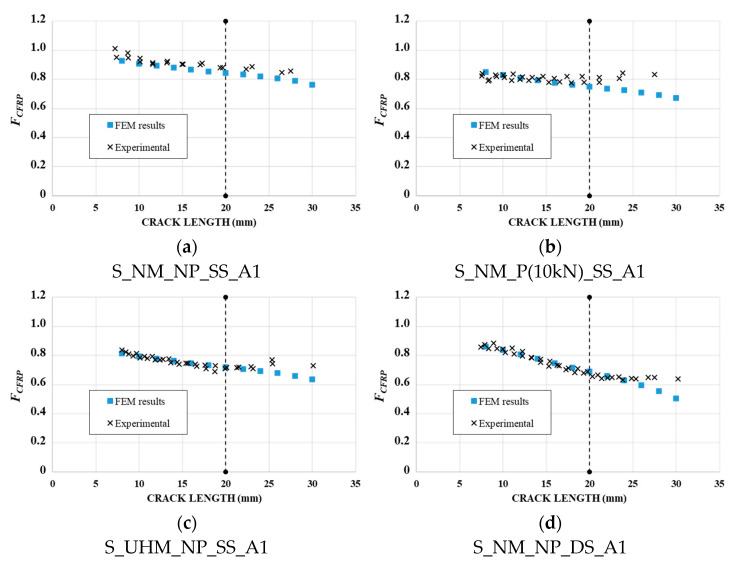
Comparison between experimental and predicted reduction factor, *F_CFRP_*, using averaged FEM results: (**a**) NM-non-pre-stressed single-sided reinforced specimen; (**b**) NM-pre-stressed single-sided reinforced specimen; (**c**) UHM-non-pre-stressed single-sided reinforced specimen; (**d**) NM-non-pre-stressed double-sided reinforced specimen.

In conclusion, the values (*K_mid*) and (*K_avg*) were considered, respectively, for single-sided and double-sided reinforced specimens, as the numerical modified SIF to use for determining the numerical reduction factor *F_CFRP_*. A comparison between the experimental and numerical reduction factor, *F_CFRP_*, is presented in Figure 13, for each reinforcement configuration. The vertical line at 20 mm represents the end of the validity of the Paris law field.

We can observe a good correlation between the experimental and numerical reduction factor for all reinforced specimens. Therefore, the selected numerical SIF values (*K_avg* for double-sided reinforced specimens and *K_mid* for single-sided reinforced specimens) seemed to be well suited in order to determine the reduction factor *F_CFRP_*.

Figure 13 clearly highlights that the SIF of the cracked specimens was efficiently reduced by applying CFRP reinforcement, a smaller reduction factor representing a better reinforcement effect of CFRP laminate.

### 4.3. Parametric Analysis—Pre-Stressing Level

The effect of the pre-stressing level of CFRP laminate was investigated using the previous finite element model. Five levels of pre-stressing force in the CFRP laminate were considered: 10, 20, 30, 40 and 50 kN. The pre-stressing force was set using thermal boundary condition for the CFRP laminate. The linear thermal expansion of the CFRP laminate was considered in the material data and different negative temperatures were set to the CFRP laminate corresponding to each pre-stressing level.

**Figure 14:**
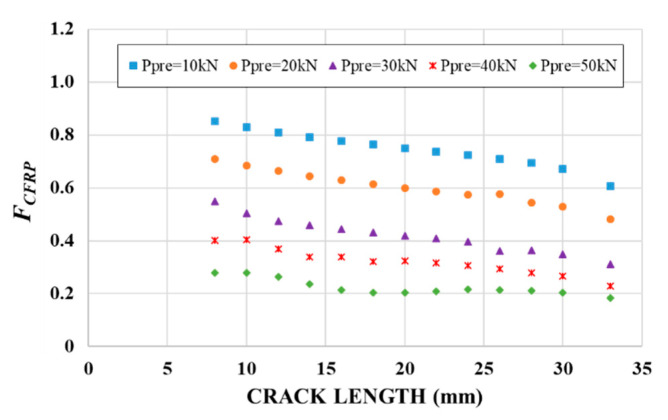
Evolution of the reduction factor *F_CFRP_* with the pre-stressing force of the CFRP laminate.

Figure 14 presents the reduction factor versus crack length obtained for the five pre-stressing levels. As expected, it illustrates a substantial reduction in the SIF as the pre-stressing level increased, which will result in an increase of the fatigue life of the element.

**Figure 15:**
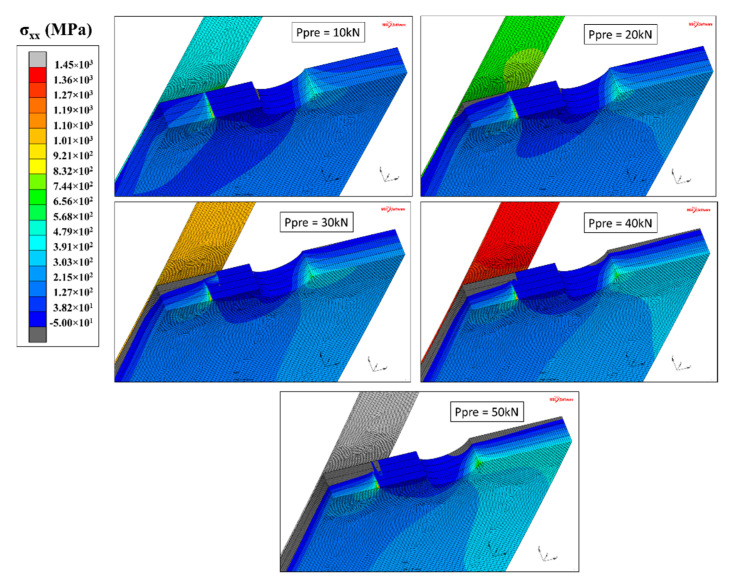
Compressive stress state in the steel plate according to the pre-stressing force in the CFRP laminate.

As the pre-stressing force increased, the *F_CFRP_*-a curve starts to flatten. This was due to the compressive stress that took place in the thickness of the steel plate, as shown in Figure 15.

We can observe that a higher pre-stressing level provided higher compressive stresses (gray area in Figure 15). For specimens with pre-stressing force higher than 20 kN, elements directly under the patched side remained in compression throughout the loading period. Nevertheless, due to the un-symmetrical reinforcement configuration (causing a significant bending moment in the steel plate proportional to the pre-stressing level), this compressive stress state could not take place throughout the thickness of the plate. Thus, in all cases, the elements at the un-patched side remained in tension and the crack continued to propagate, but at a lower rate.

## 5. Conclusions

A finite element model was developed to study the fatigue crack propagation in cracked steel plates repaired with various CFRP bonding configurations. The FEM results were compared with the experimental ones in order to assess the accuracy of the model. The obtained results led to the following conclusions:

- Good fitting was observed between experimental and numerical results. The proposed FEM can thus be successfully used in order to evaluate the fatigue behavior of repaired cracked steel plate by CFRP bonding.
- Single-sided CFRP reinforcement led to a non-uniform distribution of the SIF along the crack front. A substantial decrease of SIF was observed immediately under the patched surface, and even more for specimens reinforced by UHM CFRP laminate and pre-stressed NM CFRP laminate. Therefore, for this reinforcement configuration, it appears essential to identify the adequate SIF value to consider for each crack front. Comparison between experimental and numerical results showed that the use of the SIF value at mid-thickness of the plate allows a good assessment of the fatigue life.
- Double-sided reinforced specimens exhibited a uniform distribution of the SIF along the crack front, resulting in a better increase in fatigue lifetime compared to single-sided reinforced specimens. For this reinforcement configuration, the average value of SIF, for each crack front, can be used to assess the fatigue life.
- By considering the reduction factor *F_CFRP_* in the closed-form solution of SIF, the mode-I SIF for CFRP-repaired specimens can be obtained, taking into account both the effects of the mechanical properties of CFRP laminates (CFRP modulus, application of pre-stressing force) and the reinforcement geometry (single-sided or double-sided bonding).
- The parametric study regarding the effect of pre-stressing level for single-sided reinforced specimens showed that a higher pre-stressing level led to a greater reduction in SIF directly under the patched surface. A bigger compressive stress zone through thickness was also observed for higher pre-stressing level but remained limited by the un-symmetrical configuration (secondary bending).

## Figures and Tables

**Table 1:** Measured properties of metallic, carbon fiber reinforced polymer (CFRP) and adhesive materials.

Material Properties	S235 Carbon Steel	NM CFRP Laminate	UHM CFRP Laminate	Adhesive A	Adhesive B
Tensile strength (MPa)	506 (3.40)	-	-	21.4 (2.96)	29.5 (0.75)
Yield strength (MPa)	250 (14.50)	-	-	-	-
Modulus (MPa)	197,000 (2100)	165,000	460,000	3650 (290)	2400 (56)
Thickness (mm)	10	1.2	2.3	0.5	0.5

The standard deviation is indicated in brackets.

**Table 3:** Input mechanical properties in finite element model (FEM) for isotropic materials.

Material	$E_{x}\left(MPa\right)$	$E_{y,z}\left(MPa\right)$	$\nu_{xy,xz,yz}$	$\nu_{yz}$	$\nu_{zx}$	$G_{xy,xz}\left(MPa\right)$	$G_{yz}\left(MPa\right)$
Steel plate	200,000	-	0.3	-	-	-	-
NM CFRP laminate	165,000	9100	0.3	0	0.017	3400	4600
Adhesive A	3650	-	0.3	-	-	-	-
UHM CFRP laminate	460,000	9900	0.3	0	0.006	3700	5100
Adhesive B	2400	-	0.3	-	-	-	-

**Table 2:** Experimental results for reinforced S235 carbon steel specimens.

Specimen	Specimen Number	Fatigue Life (Cycles)	Fatigue Life Increase Ratio	Average Fatigue Life Increase Ratio
S_R (S235 carbon steel reference specimens)	1	429,798	-	-
	2	403,639		
S_NM_NP_SS	1	487,675	1.17	1.27 (0.064)
	2	564,600	1.35	
	3	533,086	1.28	
S_NM_P(10kN)_SS	1	770,000	1.85	1.74 (0.105)
	2	681,902	1.64	
S_NM_NP_DS	1	1,080,215	2.59	2.27 (0.170)
	2	902,460	2.17	
	3	958,183	2.3	
	4	849,870	2.04	
S_UHM_NP_SS	1	765,611	1.84	2.18 (0.227)
	2	1,013,660	2.43	
	3	944,594	2.27	

The standard deviation for fatigue life increase ratio is indicated in brackets.

## Data Availability

The data presented in this study are available on request from the corresponding author.
